# Interactive effects of vascular risk burden and advanced age on cerebral blood flow

**DOI:** 10.3389/fnagi.2014.00159

**Published:** 2014-07-07

**Authors:** Katherine J. Bangen, Daniel A. Nation, Lindsay R. Clark, Alexandrea L. Harmell, Christina E. Wierenga, Sheena I. Dev, Lisa Delano-Wood, Zvinka Z. Zlatar, David P. Salmon, Thomas T. Liu, Mark W. Bondi

**Affiliations:** ^1^Psychology Service, VA San Diego Healthcare SystemSan Diego, CA, USA; ^2^Department of Psychiatry, University of California, San DiegoLa Jolla, CA, USA; ^3^Department of Psychology, University of Southern CaliforniaLos Angeles, CA, USA; ^4^San Diego Joint Doctoral Program in Clinical Psychology, San Diego State University/University of CaliforniaSan Diego, CA, USA; ^5^Research Service, VA San Diego Healthcare SystemSan Diego, CA, USA; ^6^Department of Neurosciences, University of CaliforniaSan Diego, La Jolla, CA, USA; ^7^Department of Radiology, University of CaliforniaSan Diego, La Jolla, CA, USA

**Keywords:** aging, vascular risk factors, arterial spin labeling, cognition

## Abstract

Vascular risk factors and cerebral blood flow (CBF) reduction have been linked to increased risk of cognitive impairment and Alzheimer's disease (AD); however the possible moderating effects of age and vascular risk burden on CBF in late life remain understudied. We examined the relationships among elevated vascular risk burden, age, CBF, and cognition. Seventy-one non-demented older adults completed an arterial spin labeling MR scan, neuropsychological assessment, and medical history interview. Relationships among vascular risk burden, age, and CBF were examined in *a priori* regions of interest (ROIs) previously implicated in aging and AD. Interaction effects indicated that, among older adults with elevated vascular risk burden (i.e., multiple vascular risk factors), advancing age was significantly associated with reduced cortical CBF whereas there was no such relationship for those with low vascular risk burden (i.e., no or one vascular risk factor). This pattern was observed in cortical ROIs including medial temporal (hippocampus, parahippocampal gyrus, uncus), inferior parietal (supramarginal gyrus, inferior parietal lobule, angular gyrus), and frontal (anterior cingulate, middle frontal gyrus, medial frontal gyrus) cortices. Furthermore, among those with elevated vascular risk, reduced CBF was associated with poorer cognitive performance. Such findings suggest that older adults with elevated vascular risk burden may be particularly vulnerable to cognitive change as a function of CBF reductions. Findings support the use of CBF as a potential biomarker in preclinical AD and suggest that vascular risk burden and regionally-specific CBF changes may contribute to differential age-related cognitive declines.

## Introduction

Vascular risk factors increase risk of cognitive impairment and Alzheimer's disease (AD) (Luchsinger et al., 2005; Gorelick et al., 2011). Although it is known that the link between vascular risk and cognitive decline is independent of clinical stroke (Gorelick et al., 2011) and vascular brain lesions imaged with magnetic resonance imaging (MRI) (Zheng et al., 2012), how vascular risk might lead to increased risk of AD has yet to be determined. According to the “two-hit vascular hypothesis” of AD (Zlokovic, 2011), vascular risk factors may lead to blood–brain barrier (BBB) dysfunction and reduced cerebral blood flow (CBF), initiating a cascade of processes that lead to dementia. In the primary (non-amyloid-β) pathway (hit one), accumulation of neurotoxic molecules and capillary hypoperfusion lead to neuronal dysfunction. Vascular dysfunction also leads to increased production and decreased clearance of amyloid-β, leading to amyloid-β accumulation. This increase in amyloid-β (hit two) leads to further neuronal dysfunction, accelerating neurodegeneration, and the development of dementia. Both amyloid-β and hypoperfusion may increase hyperphosphorylation of tau thereby leading to neurofibrillary tangle formation. Vascular-mediated neuronal dysfunction may be particularly relevant for very-old adults (i.e., those over age 80) given that arterial stiffness and vascular disorders are more common and more severe in this age group (De Leeuw et al., 2001).

Evidence from neuropathologic studies suggest that the presence of cerebrovascular disease (CVD) may lower the threshold of AD pathology accumulation necessary to clinically unmask dementia (Chui et al., 2012). AD patients with comorbid CVD show less AD pathology than AD patients without CVD (Esiri et al., 1999), even when patients with and without CVD are identical in terms of level of dementia severity (Bangen et al., in press). The association between AD pathology and dementia is attenuated among very-old adults (Prohovnik et al., 2006; Savva et al., 2009) and neuropathologic studies show a greater prevalence of mixed pathology with both AD and vascular features along with fewer cases of “pure” AD pathology among the oldest-old (Jellinger and Attems, 2010). Taken together, these studies suggest that vascular dysfunction is an important mechanism leading to cognitive impairment (Pantoni, 2010), and may play an even more prominent role among very-old adults.

Arterial spin labeling (ASL) MRI has been employed to reliably measure CBF across the aging-MCI-AD continuum (Johnson et al., 2005; Restom et al., 2007; Bangen et al., 2009). Abnormal resting state CBF may be an early indicator of brain dysfunction in individuals at risk for developing dementia (Fleisher et al., 2009; Bangen et al., 2012; Wierenga et al., 2012). ASL studies of dementia and at-risk populations demonstrate patterns of regional hypoperfusion similar to those revealed by positron emission tomography (PET) and single photon emission computed tomography (SPECT) (Detre and Alsop, 1999; Alsop et al., 2000). Advantages of ASL over PET and SPECT include the use of an endogenous tracer (rather than an intravenously administered contrast agent), relatively brief scan times (typically 5–10 min), and the ability to provide dynamic CBF estimates (with a temporal resolution on the order of seconds) due to the rapid decay of the tracer (Johnson et al., 2005). Given these factors, ASL MRI may provide a sensitive technique for identifying at-risk individuals, monitoring changes in neural activity due to developing neuropathology, and assessing effectiveness of disease-modifying treatments.

Although associations have been consistently found between aging and hypoperfusion and between vascular risk factors and hypoperfusion (Grolimund and Seiler, 1988; Bangen et al., 2009; Muller et al., 2012), the *interaction* between advanced age and vascular risk burden on CBF remains unclear. Furthermore, most studies have focused on individual vascular risk factors, however, multiple vascular risk factors often co-exist (Genest and Cohn, 1995) and have been shown to incrementally increase risk for AD (Luchsinger et al., 2005; Whitmer et al., 2005). Studies often examine individual risk factors while adjusting for additional risk factors, but this approach may lead to over-adjustment and underestimation of effects (Szklo and Nieto, 2000; Luchsinger et al., 2005). Our previous research demonstrated that aggregate vascular risk in particular was associated with CVD in a sample of autopsy-confirmed AD patients, highlighting the potential importance of aggregate risk in the vascular contribution to cognitive impairment (Bangen et al., in press).

Therefore, the present study aimed to elucidate the relationships among elevated vascular risk burden, age, CBF, and cognition. We predicted that the presence of elevated vascular risk burden (i.e., multiple vascular risk factors) would interact with advancing age to result in reduced CBF. CBF was measured in regions of interest (ROIs) that were selected based on previous results suggesting their vulnerability to small vessel disease and association with AD and aging. We further predicted that reduced CBF in specific ROIs would correlate with poorer neuropsychological performance in associated cognitive domains [e.g., reduced CBF in medial temporal (MTL) regions would be associated with poorer memory]. Finally, we predicted that, those older adults with elevated vascular risk burden would be particularly vulnerable to cognitive change as a function of CBF reduction. Such findings may improve detection of individuals at risk for cognitive impairment and may help inform the development of treatments designed to slow or prevent cognitive decline.

## Materials and methods

### Participants

Seventy-one independently living, non-demented older adults were recruited from the San Diego community and ongoing studies at the University of California San Diego (UCSD) Shiley-Marcos Alzheimer's Disease Research Center (ADRC). Potential participants were excluded if they were younger than 65 years of age; had dementia identified by medical, neurological, and neuropsychological examinations; or had a history of neurologic disease, head injury with loss of consciousness, learning disability, or major psychiatric disorder. Of the 71 participants, two had three vascular risk factors (hypertension, diabetes, cardiovascular disease, atrial fibrillation, history of transient ischemic attack [TIA]/minor stroke, or current smoking), 14 had two vascular risk factors, 33 had one vascular risk factor and 22 had none. For analytical purposes, those with multiple (i.e., two or three) vascular risk factors were collapsed into one category and compared to those with no or one vascular risk factor. Thus, we compared higher and lower vascular risk burden groups. All data were collected in accordance with UCSD and VA San Diego Healthcare System institutional review board-approved procedures and within the guidelines of the Helsinki Declaration. All participants provided written informed consent prior to enrollment.

### Clinical and neuropsychological assessment

All participants underwent a semi-structured interview regarding medical and psychiatric history; neurological examination; assessment of functional abilities; physical examination with brachial artery blood pressure measurement using an automated blood pressure cuff; neuropsychological testing; buccal swab DNA extraction for APOE genotyping; and brain MRI. The presence or absence of vascular risk factors derived from the Framingham Stroke Risk Profile (D'Agostino et al., 1994) was determined by self-report, medical chart review, and physical examination. Targeted vascular risk factors included: (1) hypertension; (2) diabetes; (3) history of cardiovascular disease (e.g., coronary artery disease (myocardial infarction, angina pectoris, coronary insufficiency), intermittent claudication, cardiac failure); (4) atrial fibrillation; (5) TIA or minor stroke; and (6) current smoking. Hypertension was defined as systolic blood pressure ≥ 140 mm Hg, diastolic blood pressure ≥ 90 mm Hg, or use of antihypertensive medications. Each vascular risk factor was assigned a value of 0 if absent and 1 if present. Global cognition was assessed by the Dementia Rating Scale (DRS) (Mattis, 1988), episodic memory was assessed by the California Verbal Learning Test-Second Edition (CVLT-II) (Delis et al., 2000), and executive functioning was assessed by Part B of the Trail Making Test (one participant in the high vascular risk group did not complete the Trail Making Test). APOE genotype was determined using a polymerase chain reaction-based method (Saunders et al., 1993).

### MRI acquisition

Participants were scanned on a GE Signa HDx 3.0 Tesla whole body MR scanner using an 8-channel receive-only head coil (General Electric Medical Systems, Milwaukee, WI, USA). A T1-weighted anatomical scan was acquired at 1 mm^3^ resolution using either a 3D MPRAGE sequence (26 cm FOV, 256 × 256 matrix, *TR* = 7 ms, *TE* = 3 ms, flip angle = 8°, inversion time = 900 ms, bandwidth = 31.25 kHz, and 170 1.2 mm sagittal slices) or a 3D FSPGR sequence (identical parameters to MPRAGE except 25 cm FOV, 256 × 192 matrix, *TR* = 8.1 ms, inversion time = 600 ms, 172 1 mm sagittal slices). T2-weighted fluid attenuated inversion recovery (FLAIR) images (20 cm FOV, 256 × 256 matrix, flip angle = 90°, *TE* = 142 ms, *TR* = 10000 ms, 5 mm axial slices with no interslice gap) were acquired for a subset of 41 participants (35 with low and 6 with high vascular risk burden). A resting state pulsed ASL scan was acquired using a modified flow-sensitive alternating inversion recovery sequence (Kim, 1995) [post-saturation and inversion times of TI1 = 600 ms and TI2 = 1600 ms, *TR* = 2500 ms, *TE* = 3.2 ms FOV = 22 × 22 cm, 64 × 64 matrix, 20 5 mm axial slices, 40 volumes (20 tag+control image pairs)]. This sequence utilized presaturation pulses and PICORE QUIPSS 2 post-inversion saturating pulses and a spiral read out with four interleaves (Wong et al., 1998). A scan with the inversion pulses turned off was acquired to obtain an estimate of the magnetization of cerebrospinal fluid (CSF). The CSF signal was used to estimate the equilibrium magnetization of blood, which was used to convert the perfusion signal into calibrated CBF units (millimeters of blood per 100 g of tissue per minute) (Chalela et al., 2000). In addition, a minimum contrast scan was acquired to adjust for coil inhomogeneities during the CBF quantification step (Wang et al., 2005).

### MRI data processing

MRI data were processed using Analysis of Functional NeuroImages (AFNI) (Cox, 1996), FMRIB Software Library (FSL) (Smith et al., 2004), and locally created MATLAB scripts.

#### T1-weighted anatomical images

Following N3 bias correction of field inhomogeneities, structural scans were skull-stripped using Brain Surface Extractor (Version 3.3) (Shattuck et al., 2001), an approach shown to be very effective when working with the images of older adults (Fennema-Notestine et al., 2006). Scans were manually edited when necessary to remove any residual non-brain material remaining after the automated skull stripping. Whole brain images were then segmented into gray matter (GM), white matter (WM), and CSF compartments using FSL's FMRIB's Automated Segmentation Tool (FAST) (Zhang et al., 2001). High-resolution anatomical images and partial volume segmentations were registered to ASL space and down-sampled to the resolution of the ASL image using AFNI.

#### T2-weighted FLAIR images

Although CBF is of primary interest, we assessed white matter lesions (WML) given evidence linking them to advanced aging and vascular risk (Raz et al., 2012). For quantification of WML volume, we applied a semiautomated volumetric approach to T2-FLAIR images using a reliable, previously published method (Delano-Wood et al., 2008). This type of semi-automated volumetric approach is a methodology shown to be the most reliable approach for the analysis of WML when compared to other image types and traditional quantitative visual rating scales (Price et al., 2005). Briefly, using AFNI, circumscribed areas of increased signal intensity within the WM were manually traced in 17–21 image slices per participant in the axial plane. WML volume was quantified as the total number of voxels (mm^3^) of these hyperintense regions.

#### ASL images

Each ASL dataset was reconstructed using the SENSE algorithm (Pruessmann et al., 1999). To minimize effects of participant motion, the ASL time series was co-registered to the middle time-point. A mean ASL image was formed for each participant from the average difference of control and tag images using surround subtraction. Slice timing delays were accounted for to ensure the inversion time (TI2) was slice specific (Liu and Wong, 2005). The mean ASL image was converted to absolute units of CBF (milliliters per 100 g of tissue per minute) using the CSF image as a reference signal (Chalela et al., 2000). To correct CBF data partial volume effects and minimize the effects on the CBF estimates of the lower perfusion in WM and the lack of perfusion in CSF, we used a previously published method that assumes that CSF has zero CBF and that CBF in GM is 2.5 times greater than in WM (Johnson et al., 2005). Partial-volume-corrected CBF signal intensities were calculated using the following formula: CBF_corr_ = CBF_uncorr_/(GM + 0.4 ^*^ WM). CBF_corr_ and CBF_uncorr_ are corrected and uncorrected CBF values, respectively. GM and WM are GM and WM partial volume fractions, respectively, and were computed based on the tissue content of each perfusion voxel as determined by FSL's FAST program. The CBF_corr_ data were spatially smoothed to a resolution of 4 mm full-width at half-maximum. CBF voxels with negative intensities were replaced with zero (Brown et al., 2003). In addition, we used a conservative threshold that removed CBF values outside of the expected physiological range of CBF (below 10 or greater than 150) from analyses. CBF data were normalized to the atlas of Talairach and Tournoux (1988) and re-sampled at a 4 mm^3^ resolution.

### Statistical analyses

#### Demographic and clinical data

Independent samples *t*-tests for continuous variables and χ^2^ tests for categorical variables were used to compare the participant groups on demographic and clinical variables.

#### Neuroimaging data

ROI analyses for the CBF data included two subcortical and four cortical regions defined in AFNI using the Talairach atlas. The two subcortical ROIs, caudate and thalamus, were selected because of their vulnerability to small vessel disease. The four cortical ROIs were MTL (hippocampus, parahippocampal gyrus, uncus), inferior parietal (supramarginal gyrus, inferior parietal lobule, angular gyrus), posteromedial (posterior cingulate, precuneus, cuneus), and frontal (anterior cingulate, middle frontal gyrus, medial frontal gyrus) cortices. These four cortical regions were selected because MTL, inferior parietal, and posteromedial cortices have been implicated in early AD, and frontal lobe has been associated with aging and elevated vascular risk. The average quantified CBF corrected for partial volume effects was extracted for each of the six ROIs. Hierarchical multiple regression analyses were performed to investigate the interaction between age and vascular risk burden (high and low) in each ROI. All models included sex and APOE ε4 status (ε4 carrier or non-carrier) as covariates in the first block, main effects of age and vascular risk burden in the second block, and age by vascular risk burden interaction terms in the third block.

Associations between cognitive performance and CBF in ROIs with significant interactions between age and vascular risk burden were examined for each subgroup (high and low vascular risk burden) with bivariate correlations. Performance on the CVLT-II (List A Trials 1–5 raw score) was correlated with CBF in MTL and posteromedial ROIs given the role of these regions in episodic memory. Performance on Part B of the Trail-Making Test (total time raw score) was correlated with CBF in frontal and parietal ROIs given the role of these regions in executive functioning and visual attention/spatial cognition/visuomotor integration. Significance levels of 0.05 were used for all tests. All analyses were performed using SPSS (version 18.0).

## Results

### Demographic and clinical characteristics

Participants with low and high vascular risk burden did not significantly differ in terms of mean age, years of education, sex distribution, global cognitive functioning as assessed by the DRS, depressive symptomatology, or APOE genotype (all *p*-values > 0.05; Table 1). As expected, the group with high vascular risk burden had significantly greater frequencies of many of the vascular risk factors including hypertension, diabetes, cardiovascular disease, and history of TIA or clinical stroke. In addition, compared to those with low vascular risk burden, they had significantly reduced diastolic blood pressure, which may result from atherosclerosis and arterial stiffening (Qiu et al., 2005), and they were also more likely to be taking anti-hypertensive medication (see Table 1).

**Table 1:** **Demographic and clinical characteristics of participants**.

	**Low vascular risk Mean (*SD*)**	**High vascular risk Mean (*SD*)**	***t* or χ^2^**	***P***
*N*	55	16		
Age (years)	74.73 (7.92)	75.94 (7.35)	0.55	0.59
Education (years)	16.31 (2.41)	15.13 (2.03)	1.79	0.08
Women/men (% Women)	33/22 (60.0%)	8/8 (50.0%)	0.51	0.48
APOE ε4 carrier/non-carrier (% ε4 carrier)^*^	16/37 (29.1%)	9/7 (56.3%)	3.61	0.06
DRS total score	139.82 (3.94)	139.56 (4.50)	0.22	0.83
CVLT-II trials 1–5 total (*T*-score)	46.43 (11.87)	46.13 (11.74)	0.43	0.67
Trails B (s)	80.57 (29.81)	78.67 (34.19)	0.21	0.83
Geriatric depression scale	3.40 (3.82)	4.27 (3.54)	0.79	0.43
Systolic blood pressure	129.16 (16.21)	130.06 (12.45)	0.21	0.84
Diastolic blood pressure	77.05 (9.30)	71.31 (8.26)	2.23	0.03
Use of antihypertensive medications, *n* (%)	23 (41.8%)	14 (87.5%)	10.39	0.006
Hypertension, *n* (%)	27 (49.1%)	15 (93.8%)	10.23	0.001
Diabetes, *n* (%)	1 (1.8%)	4 (25%)	10.18	0.001
Cardiovascular disease, *n* (%)	0	7 (43.8%)	26.69	<0.001
History of atrial fibrillation, *n* (%)	3 (5.5%)	3 (18.8%)	2.83	0.09
Current smoker, *n* (%)	2 (3.6%)	0	0.60	0.44
History of TIA or stroke, *n* (%)	0	5 (31.3%)	18.49	<0.001

SD, standard deviation; APOE, apolipoprotein E; DRS, Mattis Dementia Rating Scale; CVLT-II, California Verbal Learning Test-Second Edition.

* Two participants with low vascular risk factors burden were missing APOE genotype data.

### Cerebral blood flow: interaction of vascular risk burden and age

**Figure 1:**
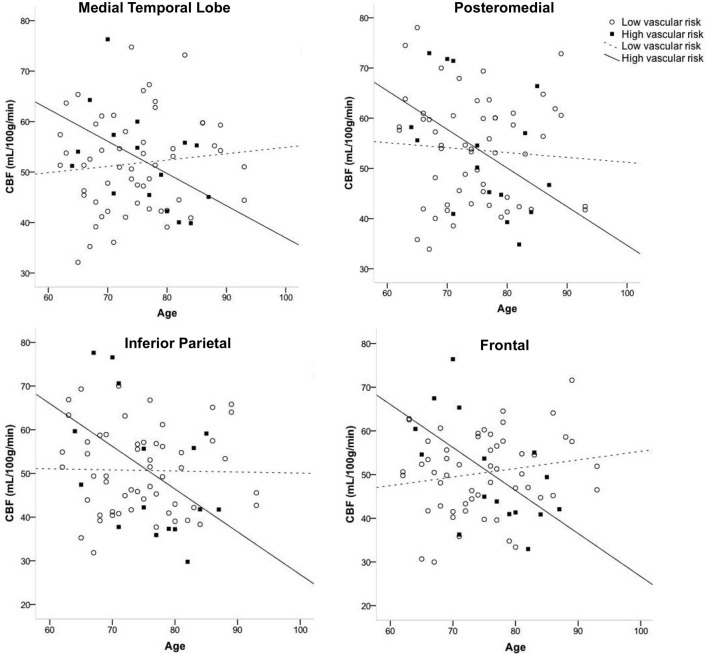
**Interaction of age and vascular risk burden on cerebral blood flow (CBF) for four *a priori* cortical regions of interest**. Interactions were statistically significant for the medial temporal, inferior parietal, and frontal regions of interest (*p*-values < 0.05) and there was a trend toward an interaction for posteromedial CBF (*p* = 0.12). High vascular risk indicates the presence of two or more vascular risk factors whereas low vascular risk indicates the presence of no or one vascular risk factor.

Figure 1 displays the average quantified CBF (corrected for partial volume effects) in each of the four cortical ROIs as a function of age and vascular risk burden. Multiple regression analyses showed that, after adjusting for sex and APOE ε4 status, there were significant interactions between age and vascular risk burden for the MTL (*R*^2^ = 0.07, *B* = −0.86, *p* = 0.02), inferior parietal (*R*^2^ = 0.07, *B* = −1.05, *p* = 0.02), and frontal ROIs (*R*^2^ = 0.11, *B* = −0.66, *p* = 0.002) and a trend toward a significant interaction for the posteromedial ROI (*R*^2^ = 0.03, *B* = −0.63, *p* = 0.12). As illustrated in Figure 1, the interaction effects were characterized by a negative relationship between age and CBF in the high vascular risk burden group but no such relationship in the low vascular risk burden group. Follow up bivariate correlational analyses were conducted for those with low and high vascular risk burden separately. These follow up analyses demonstrated that, among those with high vascular risk burden, aging was associated with significantly reduced CBF or trends toward significantly reduced CBF across all four ROIs (MTL CBF: *r* = −0.49, *p* = 0.06; Posteromedial CBF: *r* = −0.46, *p* = 0.08; Inferior parietal CBF: *r* = −0.48, *p* = 0.06; Frontal CBF: *r* = −0.59, *p* = 0.01). In contrast, among those with low vascular risk burden, there were no significant associations or trends toward significant associations between age and CBF (MTL CBF: *r* = 0.10, *p* = 0.45; Posteromedial CBF: *r* = −0.07, *p* = 0.60; Inferior parietal CBF: *r* = −0.02, *p* = 0.89; Frontal CBF: *r* = 0.17, *p* = 0.21). There were no significant interactions between age group and vascular risk status for the two subcortical regions: thalamus (*R*^2^ = 0.02, *B* = −0.75, *p* = 0.18) and caudate (*R*^2^ = 0.008, *B* = −0.28, *p* = 0.42). There were no main effects of age or vascular risk status on CBF across any of the six ROIs (*p*-values > 0.05 see Table 2).

**Table 2:** **Main and interaction effects of age and vascular risk burden on CBF**.

**Block**	**Variable**	**Medial temporal CBF**		**Inferior parietal CBF**		**Posteromedial CBF**		**Frontal CBF**		**Thalamus CBF**		**Caudate CBF**	
		**Δ*R*^2^**	***B* (*SE*)**	**Δ*R*^2^**	***B* (*SE*)**	**Δ*R*^2^**	***B* (*SE*)**	**Δ*R*^2^**	***B* (*SE*)**	**Δ*R*^2^**	***B* (*SE*)**	**Δ*R*^2^**	***B* (*SE*)**
1	Sex	0.06	7.41 (3.80)	0.10^*^	12.30^**^ (4.68)	0.16^**^	14.53^***^ (4.04)	0.18^***^	8.44^***^ (2.22)	0.19^**^	22.68^***^ (5.74)	0.19^***^	12.68 (3.5)^***^
	APOE ε4 status		2.00 (3.96)		4.54 (4.88)		0.86 (4.2)		−0.59 (2.3)		5.68 (5.99)		6.42 (3.65)
2	Age	0.01	0.16 (0.25)	<0.001	−0.05 (0.31)	0.003	−0.12 (0.27)	0.003	0.03 (0.15)	0.03	−0.57 (0.37)	0.04	−0.22 (0.27)
	Vascular risk		2.34 (4.68)		0.46 (5.79)		0.67 (4.50)		1.16 (2.74)		4.78 (6.97)		6.83 (4.23)
3	Age × vascular risk	0.07^*^	−0.86 (0.37)	0.07^*^	−1.05^*^ (0.45)	0.03	−0.63 (0.40)	0.11^**^	−0.66 (0.21)^**^	0.02	−0.75 (0.56)	0.008	−0.28 (0.34)

* *p < 0.05*,

** *p ≤ 0.01*,

*** p ≤ 0.001.

B, unstandardized coefficient estimate.

In the subset of participants with T2-FLAIR imaging, hierarchical regression models adjusting for sex and APOE ε4 status showed that there was no interaction between age and vascular risk burden on total WML volume (*R*^2^ = 0.005, *B* = −139.19, *p* = 0.54). Furthermore, there was no main effect of vascular risk burden on total WML volume (*B* = −1211.40, *p* = 0.48). There was, however, a main effect of age, with advancing age associated with greater total WML volume (β = 0.32, *p* = 0.02). WML volume was not significantly correlated with CBF in any ROI (*p*-values > 0.05).

### Association between CBF and cognition

**Figure 2:**
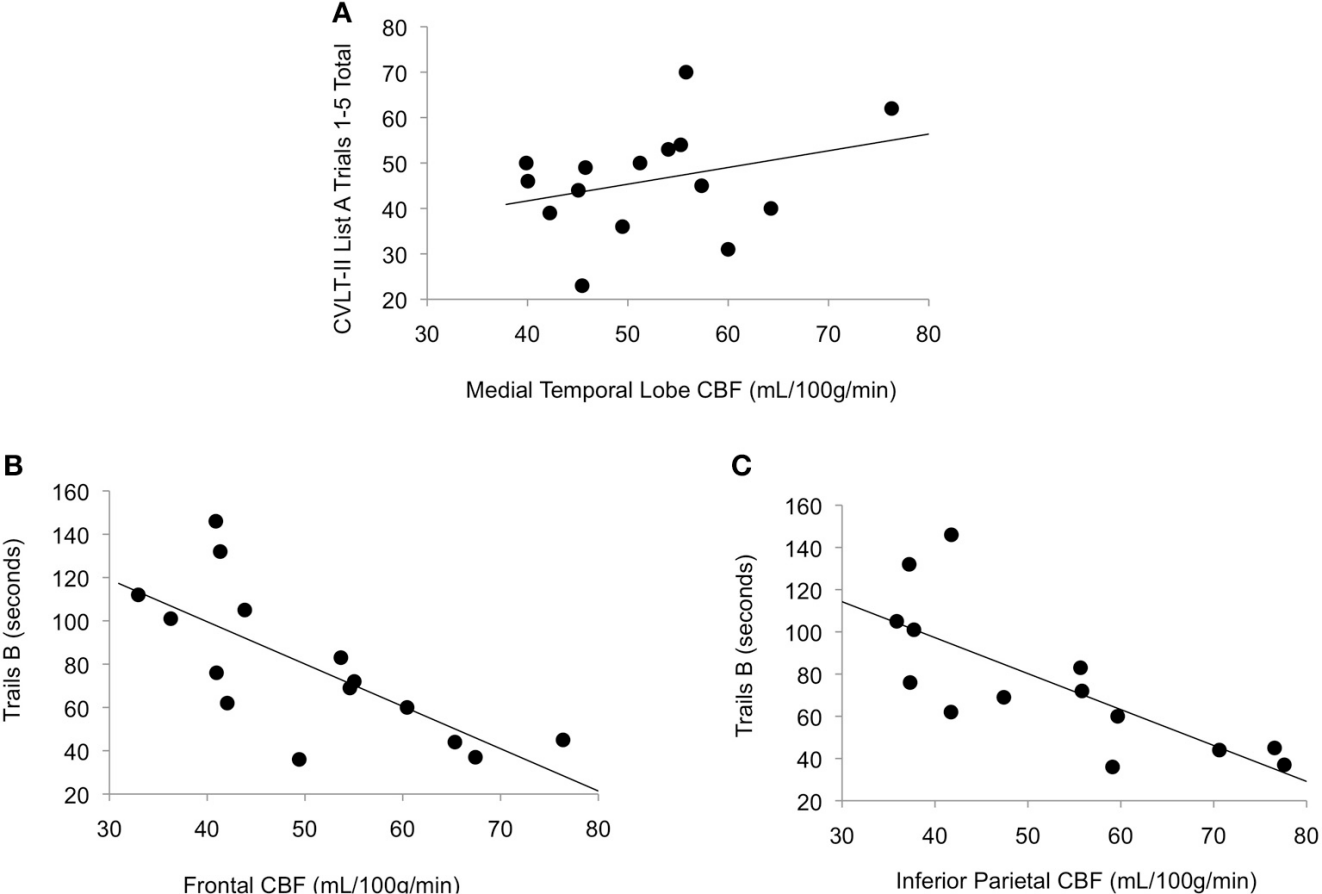
**Scatterplots of correlations between cerebral blood flow and cognitive performance for older adults with elevated vascular risk burden. (A)** Correlation between memory performance and medial temporal lobe cerebral blood flow (*p* = 0.13), **(B)** Correlation between executive function performance and frontal cerebral blood flow (*p* = 0.001), and **(C)** Correlation between executive function performance and inferior parietal cerebral blood flow (*p* < 0.001). Higher scores on Trails B represent poorer (slower) performance.

As shown in Figure 2, among those with high vascular risk burden, reduced CBF in inferior parietal (*r* = −0.77, *p* < 0.001) and frontal (*r* = −0.72, *p* = 0.001) ROIs was associated with poorer performance on an executive functioning measure requiring visual attention, spatial perception, and visuomotor integration. Also, among this group, there was a trend toward reduced CBF in MTL being associated with poorer memory performance (*r* = 0.31, *p* = 0.13). Among those with low vascular risk burden, although there was a trend toward a significant association between inferior parietal CBF and Trails B performance (*r* = 0.19, *p* = 0.09), there were no significant relationships between cognitive performance and CBF in this group (frontal CBF and Trails B: *r* = 0.10, *p* = 0.24; MTL CBF and CVLT: *r* = −0.13, *p* = 0.17).

## Discussion

The present study extends previous cerebral perfusion studies of dementia risk by demonstrating an interaction between advancing age and vascular risk burden on CBF. We found that, among those with elevated vascular risk burden, advancing age was associated with reduced CBF, whereas for the low vascular risk burden group, there was no such relationship.

This pattern was observed for cortical ROI that have been implicated in aging and AD, namely, MTL, inferior parietal, and frontal cortices. In addition, reduced CBF was associated with poorer cognitive performance in participants with elevated vascular risk burden, and this relationship was not seen in participants with low vascular risk burden, suggesting that older adults with multiple vascular risk factors may be particularly vulnerable to cognitive change as a function of CBF reduction.

Regional decreases in CBF have often been interpreted as a reflection of decreased brain function whereas increases in perfusion have frequently been interpreted as a cellular and vascular compensatory response to pathologic changes (Dai et al., 2009; Bangen et al., 2012; Wierenga et al., 2012). It has been suggested that advancing age increases risk for dementia via its tendency to reduce CBF (De La Torre, 2012a,b). Studies have reported that aging may account for an approximately 0.45–0.50% reduction in CBF per year (Leenders et al., 1990; Parkes et al., 2004). Therefore, the presence of vascular risk factors may add to the already diminished CBF that results from aging and these two burdens on CBF could decrease neuronal energy thereby leading to cognitive decline (De La Torre, 2012a). The findings of the present study suggest that the presence of multiple vascular risk factors and advanced age interact to impart more detrimental effects on CBF than either one alone.

Among those with high vascular risk burden, younger age was associated with higher CBF. In addition, greater CBF in frontal and posterior regions was associated with better executive function among those with elevated vascular risk. Taken together, these findings raise the possibility that elevated CBF may represent a compensatory mechanism in this group. It is possible that older adults with multiple vascular risk factors are able to invoke compensatory mechanisms at younger ages. However, as they age, they may have less capacity for compensation given that their brain perfusion may be already reduced due to advanced age. Burgeoning evidence suggests that cerebral autoregulation is unlikely to reverse brain hypoperfusion if cardiac output is compromised (De La Torre, 2012a). Therefore, the presence of cardiovascular pathology in older adults may have long-term effects on CBF because it may limit neuronal responses from being able to maintain normal regulatory and/or compensatory capabilities.

The present findings are consistent with our previous reports suggesting that multiple dementia risk factors interact to reduce CBF in regions vulnerable to early AD and aging (Wierenga et al., 2012) and may be valuable for characterizing changes in structure-function relationships in individuals at risk. We observed interactions between age and vascular risk burden on CBF in cortical regions implicated in early AD or aging (Braak and Braak, 1991, 1996; Raz et al., 1997; McDonald et al., 2009), but no interactions in subcortical regions. Notably, amyloid burden has been shown to increase over time in non-demented older adults in the posterior cingulate, frontal, parietal, and temporal cortical regions in which we observed interactions with CBF (Rowe et al., 2007; Aizenstein et al., 2008; Villemagne et al., 2008). Although conflicting reports exist regarding whether vascular risk factors directly increase AD pathology (Chui et al., 2012), some evidence links vascular dysfunction to the development of AD pathology (Altman and Rutledge, 2010) and to the accumulation of amyloid in particular (Craft, 2009; Zlokovic, 2011; Reed et al., 2012). Indeed, the “two-hit vascular hypothesis” of AD (Zlokovic, 2011) suggests that the presence of vascular risk factors initiates a cascade of events involving BBB dysfunction, hypoperfusion, and impaired clearance and accumulation of amyloid, thereby leading to dementia. Given that we did not collect markers of amyloid deposition or BBB integrity as part of this study, we cannot directly examine these mechanisms. However, regardless of the precise mechanism, the present findings suggest that, as non-demented older adults age, the presence of multiple vascular risk factors may influence neuronal function within areas that are vulnerable to early AD (Braskie et al., 2010; Bangen et al., 2012; Beason-Held et al., 2012).

In contrast to some previously published studies (Beason-Held et al., 2007; Bangen et al., 2009), we did not observe main effects of age or vascular risk factors on CBF (cf., Glodzik et al., 2011). However, these previous studies often focused on longitudinal change rather than cross-sectional group differences in CBF (Beason-Held et al., 2007, 2012), and compared young and old adults (Bangen et al., 2009; Wierenga et al., 2013) rather than the two groups of older adults included in the present study. WML volume was not significantly correlated with CBF in the current sample even though it is thought that hypoperfusion may lead to the development of WML (Zlokovic, 2011). Furthermore, there was no interaction between age and vascular risk on total WML volume. Given that hypoperfusion and the development of WML may be on a continuum, it is possible that older adults with multiple vascular risk factors who are demonstrating reduced CBF have not yet developed increased WML volume but may do so over time as chronic hypoperfusion worsens.

Several limitations should be considered when interpreting the present findings and should be addressed in future studies. As is commonly the case in neuroimaging studies, our sample size was relatively small which may have attenuated our ability to detect some group differences. It should be noted, however, that significant interactions between age and vascular risk burden on CBF were found even in this small sample with relatively low vascular risk burden. In addition, our sample was relatively well educated and medically healthy which may reduce the generalizability of the present results. Vascular risk factors in this study were categorized dichotomously as being either present or absent and vascular risk burden was characterized as high or low, rather than considering vascular risk factors as continuous variables based on risk factor severity. Further, given the cross-sectional nature of the study, causality of the relationships among aging, vascular risk, and CBF cannot be inferred. We identified hypertension based in part on the use of anti-hypertensive medications, however, it is unclear whether use of anti-hypertensive medications imparts risk or is protective (Beishon et al., 2014). Limitations of ASL MRI techniques more generally include relatively low signal-to-noise ratios and reliance on assumptions in the perfusion quantification (e.g., transit delay). The use of partial volume corrected quantitative ASL data was a strength of the study. Despite these limitations, the present study is one of the only attempts to assess the interaction between vascular risk burden and advancing age on ASL measures of CBF and cognition, making these findings a potentially useful step toward elucidating the influence of vascular risk factors on CBF abnormalities among non-demented older adults.

The present results add to a growing body of evidence demonstrating the possible influence of vascular risk burden on functional brain changes (CBF abnormalities) that may increase risk of cognitive impairment and dementia (Tzourio et al., 2001; Bangen et al., 2012). Among older adults with multiple vascular risk factors, we found association between advancing age and reduction in CBF in cortical regions implicated in early AD. Evidence suggests that cognitive and brain changes observed in very-old AD patients are less salient than those seen in younger AD patients (Stricker et al., 2011). Thus, the inclusion of additional markers may improve our ability to predict progression to dementia in very-old adults (Stricker et al., 2011). The present findings provide support for ASL MRI as a candidate biomarker for detecting changes in the central nervous system associated with vascular risk factors, particularly in older adults with elevated vascular risk burden. Finally, the present results highlight the potential utility of interventions designed to treat reduced CBF in older adults who present with vascular risk factors. Given that many vascular risk factors such as diabetes and smoking can be treated, interventions designed to target vascular risk factors in order to maintain CBF may represent important opportunities for preventing or delaying the onset of cognitive impairment and dementia (Gorelick et al., 2011; Qiu, 2012).

### Conflict of interest statement

The authors declare that the research was conducted in the absence of any commercial or financial relationships that could be construed as a potential conflict of interest.
